# Unlocking the Power of Nanopores: Recent Advances in Biosensing Applications and Analog Front-End

**DOI:** 10.3390/bios13060598

**Published:** 2023-05-31

**Authors:** Miao Liu, Junyang Li, Cherie S. Tan

**Affiliations:** Medical College, Tianjin University, Tianjin 300072, China; 3005202010@tju.edu.cn (M.L.); ljyzy@tju.edu.cn (J.L.)

**Keywords:** nanopores, biosensors, DNA sequencing, protein sequencing, chiral molecules, transimpedance amplifiers

## Abstract

The biomedical field has always fostered innovation and the development of various new technologies. Beginning in the last century, demand for picoampere-level current detection in biomedicine has increased, leading to continuous breakthroughs in biosensor technology. Among emerging biomedical sensing technologies, nanopore sensing has shown great potential. This paper reviews nanopore sensing applications, such as chiral molecules, DNA sequencing, and protein sequencing. However, the ionic current for different molecules differs significantly, and the detection bandwidths vary as well. Therefore, this article focuses on current sensing circuits, and introduces the latest design schemes and circuit structures of different feedback components of transimpedance amplifiers mainly used in nanopore DNA sequencing.

## 1. Introduction

Nanopore sensors are a type of biosensor constructed based on nanoscale pores, which can detect and analyze biomolecules such as DNA [1,2], proteins, and peptides [3,4] when they interact with the nanopores. Nanopore sensors usually consist of a nanoscale orifice and two electrodes for the detection of molecules through the interaction between molecules and the orifice, such as translocating events [5], partially entering events [6], or molecular interactions via specific tags [7]. When molecules interact with the pore, their size, shape, or other properties create different effects, producing specific signals to determine the properties and concentrations of the molecules. Nanopore sensors have wide applications in many fields, including DNA sequencing [8], protein sequencing [9], chiral molecule discrimination [5], and the detection of heavy atoms in non-biological molecules [10]. Nanopores have demonstrated their potential in commercial sequencers [11] based on biological nanopores that have been developed. However, protein sequencing faces challenges such as translocation control, spatial structure unfolding, and amino acid discrimination [12]. The application of nanopores in the field of biosensing presents significant challenges. For instance, proteins consisting of 20 natural amino acids may require specially designed nanopores comparable to the molecule’s smallest units. More importantly, the sensing region of the nanopore needs to be carefully designed to provide the required sensitivity for the ionic current generated by the smallest distinguishable units. Current engineering methods have limitations, and exploring new directions such as de novo design of nanopores and DNA origami scaffolds is necessary to customize the shape and size of nanopores [3].

When sensing the ionic current produced by nanopores, a specialized current amplification circuit, usually a dedicated integrated circuit, is typically used. The amplitude of these currents is typically at the pA level. Therefore, the application-specific integrated circuit (ASIC) utilized for nanopore sensing employs a high-gain transimpedance amplifier (TIA) at the front end. The TIA is connected to feedback elements to form a specific circuit topology, which realizes the conversion from current to voltage. Feedback elements are mainly divided into two categories: resistive feedback and capacitive feedback. For resistive feedback, the transimpedance gain is equal to the resistance value. Because nanopore current amplification requires high gain and low input reference noise, a substantial feedback resistance is required [13], usually in the giga-ohms (GΩ) range. However, as GΩ-level feedback resistors are difficult to integrate on small chips, pseudo-resistors [14,15] and switch resistors [16] can serve as feedback elements to achieve circuit performance that Ohm’s law resistance cannot achieve. Capacitive feedback architectures are divided into the Discrete Time Approach and Continuous Time Approach. Capacitive feedback topology occupies a small integrated circuit area [17] with good noise performance, which circuit designers increasingly favor. On the other hand, TIA with capacitive feedback requires complex compensation circuits to eliminate charge accumulation across the capacitor, and both Discrete-Time (DT) and Continuous-Time (CT) methods have limitations in terms of frequency range. DT is suitable for low-frequency applications, while CT is better for high-frequency applications, which makes circuit design a trade-off between bandwidth and noise reduction, posing a challenge in nanopore preamplifier design.

In this paper, we have reviewed nanopore applications in DNA sequencing, chiral molecules, and protein sequencing, and have evaluated both biological and solid-state nanopores. Furthermore, based on the classification of feedback elements, we have summarized and commented upon the state-of-the-art design of transimpedance amplifiers for nanopore simulation front-end circuits.

## 2. Applications of Nanopores

### 2.1. DNA Sequencing

DNA sequencing technology is the fundamental technique for genetic analysis, and plays an essential role in disease diagnosis and treatment in current gene-based human medical technology. The exploration of genetic sequencing technology has never stopped. DNA sequencing has undergone three generations of development, from Sanger sequencing [18] to next-generation sequencing (NGS) [19,20], and then to the latest third-generation single-molecule sequencing technology. Due to the significant breakthrough represented by nanopore sequencing technology, scholars have called nanopore sequencing the fourth-generation sequencing technology [8]. Compared with single-molecule real-time sequencing (SMRT) [21] and Heli-Scope single-molecule sequencing technology [22], nanopore sequencing detects highly subtle changes in current when an unwound nucleic acid strand passes through a nanopore. It is developing towards high throughput [23], long read lengths, low cost, and miniaturization. Commercial portable devices have been successfully developed [1,24,25,26].

Nanopore sequencing uses a nanoscale protein pore embedded in an electrically resistant polymer membrane as a biosensor. A constant voltage drives negatively charged single-stranded DNA molecules from the negative ‘cis’ side through the nanopore to the positive ‘trans’ side [1,2]. The high speed at which free DNA molecules pass through the nanopores makes it difficult to accurately identify base information from noise, resulting in a relatively low accuracy rate in base identification. Therefore, researchers utilize DNA polymerases or helicases to control and ratchet the nucleotides through the nanopore base-by-base to regulate translocation speed [27,28,29]. Because the sensing region of a nanopore is located at its narrowest aperture, the properties of nucleobases affect the ionic current passing through. When a DNA chain passes through a nanopore, causing a change in current, this reflects the specific nucleotide sequence, and allows the determination of the arrangement of bases. Therefore, the ion current variation generated during nanopore translocation events provides information about the nucleotide sequence within the effective range of the nanopore sensor.

Nanopore sequencing technology has a wide range of applications, including genome assembly [30,31,32] and base modification detection [33]. In addition, it is demonstrating its ability in medicine [34]. Specifically, it can be used to rapidly diagnose diseases [35,36], detect pathogens [37,38,39,40], and monitor epidemics [41,42,43]. Recently, nanopore sequencing has been used for cell-free DNA (cfDNA) as a means of cancer detection [23,44]. The convenience of nanopore sequencing technology in the medical field will accelerate the development of precision medicine [45,46]. Similar to pathogen detection, nanopore sequencing can be applied to various microbial detection contexts [47,48]. The wide application of nanopore sequencing has driven the corresponding development of nanopore sequencing bioinformatics [2], enabling various novel algorithms [49,50] to be applied for processing nanopore sequencing data and improving sequencing accuracy.

### 2.2. Protein Sequencing

Nanopore research is rapidly advancing towards protein analysis and protein sequencing. Protein sequencing based on nanopores has the potential for commercialization within the next decade [9]. However, protein sequencing currently relies on methods that have existed for 20 years, namely, Edman degradation [51] and mass spectrometry [52], which cannot provide single-molecule resolution. While mass spectrometry has become the gold standard for protein sequencing, it exhibits shortcomings in detection sensitivity and dynamic detection range [9]. Alternatively, nanopore-based protein sequencing methods can read protein sequences with single-molecule resolution [9], providing new hope for protein sequencing technology.

Nanopore-based DNA sequencing generates an electric current by distinguishing the four bases passing through the sensing region. However, when used for protein sequencing, three obstacles must be overcome due to the unique characteristics of proteins as biological macromolecules. First, peptide translocation control is the main obstacle in developing a nanopore protein sequencer. Unlike uniformly charged DNA molecules, amino acid residues differ significantly in charge distribution. Therefore, the simple use of electrophoretic-driven peptides for unidirectional translocation through the nanopore is not feasible. Second, unfolding structured proteins need to be considered. Most proteins fold in their native state, and in order to enable them to efficiently undergo translocation through the nanopore, their secondary and tertiary structures must be disrupted. Then, the third process is to discriminate 20 amino acids using their ionic current signatures.

Many methods have been proposed to address the problem of unfolding structured proteins. Strong denaturants such as urea, sodium dodecyl sulfate (SDS), and guanidine hydrochloride (GdnHCl) have been used successfully to unfold and translocate proteins through solid-state nanopores [53,54,55]. Denaturing agents have been used to achieve protein translocation through biological nanopores [56,57,58]. Yu and his colleagues recently achieved unidirectional slow transport of full-length proteins through a nanopore using the chemically resistant biological α-hemolysin nanopore and a high concentration guanidinium chloride buffer [59]. Solid-state nanopores have advantages over biological nanopores in structure, showing higher stability when exposed to extreme buffer conditions (8 M urea, 6 M GdnHCl, or 1% SDS). Although high temperature can be used to unfold proteins and allow the unfolded proteins to pass through nanopores [60,61], this increases the rate of protein translocation and requires higher temporal resolution for sequencing. High voltage has been shown to assist in unfolding proteins [62,63,64], but it accelerates translocation and is only applicable to solid-state nanopores. Furthermore, electroosmotic flow (EOF) [65] has been shown to control peptide translocation and extend translocation time [12,66].

In recent years, many encouraging developments have been achieved in nanopore sensing of peptides or proteins [67,68,69,70,71,72]. Among them, the following study is the most recent towards achieving the goal of protein sequencing. Ouldali and his coworker [73] proposed an ideal method for measuring natural amino acids using aerolysin nanopores. Their method could distinguish 13 amino acids, but required the net positive charge carried by the arginine heptapeptide to ensure the unidirectional electrophoretic transport of peptide segments through the nanopore. Another limitation of this method was that each amino acid should be connected to the peptide chain. The identification of all 20 amino acids may require engineered aerolysin nanopores. Nonetheless, Ouldali et al. have taken an inspiring step forward, and in the future it may be possible to sequence the protein sequence to be tested into amino acids and connect them to a carrier peptide for sequencing. Lu et al. [74] summarized the wide range of applications based on aerosol nanopore protein sequencing and single-molecule measurements and developed mutant aerolysin nanopores. Their results have made the use of aerolysin nanopores for protein sequencing more realistic. The limitations of being bound to a carrier peptide might be broken; Spitaleri et al. [75] identified peptide sequences by simulating redesigned proteins as nanopore-sensing single amino acid shapes, highly distinguishing nine different amino acids in peptide segments. Protein capture and linear movement into nanopores are essential for protein sequencing. Si et al. [76] proposed a novel navigation method that uses the different affinities of MoS_2_ and SnS_2_ to adsorb peptides onto SnS2 nanostripes while maintaining their linear conformation. The method navigates peptides towards target nanopores by changing the direction of applied voltage. Recently, Huo et al. [77] explored whether the volume exclusion model was applicable to nanopore protein sequencing and proposed a modified induction model, which can inspire nanopore protein sequencing. Brinkerhoff and colleagues used DNA helicase to thread DNA carrier molecules and peptide chains into high-resolution MspA nanopores for detecting amino acids in short peptides, achieving high-precision protein identification through multiple readouts [78]. Their research identified the composition of amino acids and determined their sequence. However, the peptide segments were designed to have a negative charge for accessible introduction into the nanopores. If random charge distribution peptide segments are used, new methods are needed. Nonetheless, their research represents an important step towards practical protein sequencing.

Despite recent progress, the path to nanopore-based protein sensing remains rife with interesting open challenges. Thus far, it remains the case that none of the three primary requirements have been entirely resolved.

### 2.3. Chiral Molecules

The application of nanopore technology extends far beyond DNA sequencing, with chiral molecule recognition emerging as a promising new application. Chirality is a crucial component of the biological and chemical realms, representing a fascinating and sophisticated aspect of these fields. Conventional chiral separation and identification methods encompass chromatography and spectroscopy [79,80,81,82]. However, emerging nanopore technology is now penetrating this field as a novel biosensing approach.

Nanopore-based single-molecule techniques have shown promise in sensing chiral molecules by designing specific chiral environments [6,83]. Boersma et al. developed a groundbreaking method using real-time protein nanopores to distinguish between enantiomers of underivatized amino acids [83]. Their findings suggest that nanopores could convert the structural and chemical properties of enantiomers into distinct ionic current signals. In addition, Cooper et al. reported that an intrinsically chiral protein nanopore can serve as a detection element to differentiate the chirality of otherwise identical supramolecular entities [6]. However, these approaches have yet to be tested for identifying single amino acids in single peptides. Further research is needed in this area.

The orientation of sidechains is the sole difference between chiral isomers, posing a challenge for identifying amino acids in peptides with equivalent volumes using conventional nanopore-based methods. In a groundbreaking study, Wang et al. revealed that OmpF, a biological nanopore from *Escherichia coli*, can distinguish between chiral amino acids within a single peptide [79]. OmpF’s charged residues are non-uniformly distributed, producing a strong lateral electrostatic field that forces peptides to adopt specific orientations as they traverse the channel. Therefore, the distinct fluctuations in ionic current can be statistically analyzed to identify peptides containing chiral amino acids and positional isomers at the single-molecule level. The discovery of OmpF’s unique ability to discriminate between chiral isomers represents a significant breakthrough in the nanopore-based analysis of peptides and proteins. Nanopores have been shown to hold great potential for applications in protein transport. Building on biomimetic strategies, Li et al. prepared chiral nanopores modified with stereoisomers of cysteine, unveiling the crucial role of chiral gatekeeping effects in protein transport [84]. Their findings demonstrate a strong impact of chirality on protein transport, with L-cysteine-modified nanopores being more favorable for protein transport due to chiral interactions. This pioneering study presents a novel approach for elucidating the selectivity of chirality in protein transport, and offers a convenient means to investigate protein chiral separation and directional therapy through leveraging chirality.

Similarly, Yuan et al. [85] explored the utilization of Covalent Organic Frameworks (COFs) as a platform for the selective transport of amino acids in this passage, demonstrating the process using 2D mesoporous COFs decorated with β-CD. CD-COF nanochannels can generate different surface charges through selective binding with L-His, which enables chiral recognition. The ability of nanopores to discriminate chiral molecules may have significant implications for quality control in the industrial production of enantiomeric drugs. A recent study by Jia et al. [86] demonstrated the potential of using MspA nanopores modified with phenylboronic acid (PBA) paired with machine learning algorithms to identify enantiomers accurately, particularly for catecholamines. This strategy presents a promising approach for improving the detection of enantiomeric drug quality and highlights the potential of nanopore technology in advancing the field of pharmaceuticals. Overall, these studies shed light on the intriguing and intrinsic feature of chirality and its impact on biological processes.

Interestingly, biological nanopores can be used to detect the flipping of individual molecules, which is easy with the capturing ability of nanopores. Du et al. [87] used engineered MspA nanopores to dynamically observe chiral boronic acid ester molecule flipping and studied the effects of different pH values on the flipping rate.

## 3. Nanopores Overview

### 3.1. Biological Nanopores

**Figure 1:**
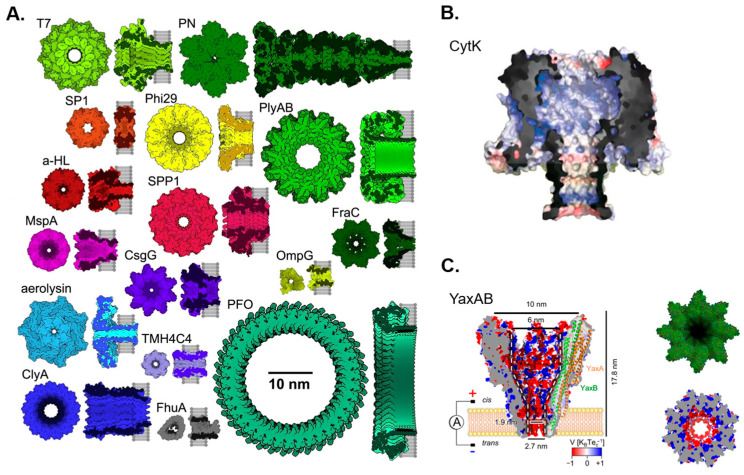
Eighteen biological nanopores used for nanopore sensing. (**A**) Sixteen biological nanopores drawn to scale. Reproduced with permission from [92]. Copyright (2022) Elsevier. (**B**) The biological nanopore Cytotoxin K (CytK). Reproduced with permission from [93]. Copyright (2022) Versloot, R.C.A. et al., published by American Chemical Society. (**C**) The biological nanopore YaxAB. Reproduced with permission from [94]. Copyright (2023) Ki-Baek Jeong et al., published by Springer Nature.

Although the idea of using nanopores for molecular detection was proposed by David Deamer in his notebook in 1989, it did not generate significant interest until Kasianowicz et al. demonstrated the detection of DNA translocation through α-hemolysin in 1996 [88]. This discovery was inspired by transporting substances across cell membranes through channel proteins, which has subsequently enlightened modern nanopore technologies. In the early days of nanopore research, most pores used were intrinsic to channel proteins, such as α-hemolysin (α-HL) [89], MspA [90], and bacteriophage phi29′s channel protein [91]. Recently, Mayer et al. summarized sixteen biological nanopores used for nanopore sensing in a review of single-molecule biological nanopore sensing [92], briefly introducing their respective properties or applications. In addition to these sixteen biological nanopores, the CytK [93] and YaxAB [94] biological nanopores have recently been used for nanopore sensing. Figure 1 displays these eighteen nanopores.

The most significant advantage of these nanopores is their remarkably unique pore structures, which can be manipulated via advanced engineering techniques [95,96] to facilitate changes in numerous aspects of the pore’s functionality. Such variations include adjusting the number of charges in the channel [97] or introducing reactive amino acids or hydrophobic groups to enhance organic molecule binding [98]. In addition, the surface charge of amino acids inside the nanopore can be modified by altering the pH, resulting in changes in electroosmotic flow [99,100,101], which is one of the advantages of biological nanopores as well.

Biological nanopores have an inherent advantage over solid-state nanopores manufactured via fabrication techniques thanks to their reproducibility and atomic-level structure. Therefore, commercially available nanopore-based DNA sequencing technology uses an array of biological nanopores [1]. Furthermore, biological nanopores exhibit a wide range of pore lumen sizes, varying from a few angstroms to several nanometers [102]. This property makes these pores highly suitable for specific sensing applications. Nevertheless, it should be highlighted that while biological nanopores are renowned for their reproducibility, they may lack the required chemical and mechanical stability to meet specific application needs [103].

### 3.2. Solid-State Nanopores

The growing interest in solid-state nanopores is attributed to their high stability and potential for large-scale production. Various materials, including silicon [104,105], silicon nitride [106], silicon oxide [105], polymers [107], aluminum oxide [108], and graphene [109,110], have been successfully utilized in nanopore fabrication. In recent studies, phosphorene [111], MoS_2_ [112,113], and MXene [112,114,115] have been found to be applicable for DNA detection and used in the production of solid-state nanopores. Generally, the process of making a nanopore involves opening and tuning methods using techniques such as FIB drilling [116], e-beam drilling [117], plasma etching [118], and wet etching [104]. In addition, additional adjustments such as deposition and thermal treatment are frequently necessary to achieve the desired pore size and shape. By combining these techniques, nanopores with various sizes, shapes, and characteristics can be generated. For example, using inorganic materials and microfabrication techniques has significantly contributed to adopting silicon nitride-based solid-state nanopores, which offer high stability and can operate under various experimental conditions involving differences in voltage, temperature, pressure, pH, and ionic strength.

**Figure 2:**
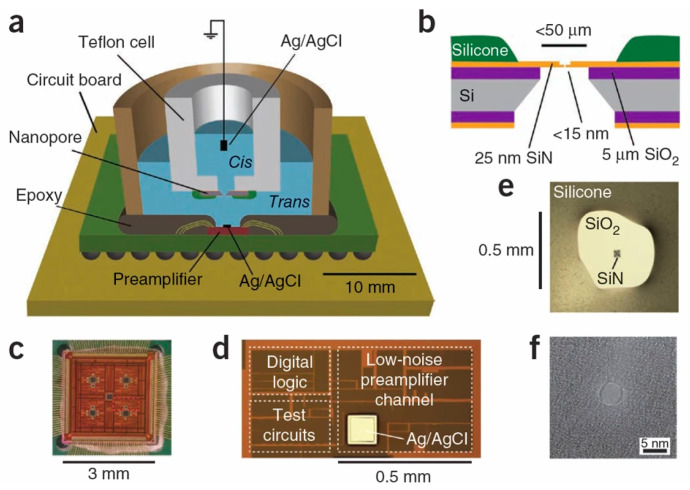
(**a**) Solid-state nanopores be integrated with front-end amplification chips. (**b**) Cross-sectional schematic of solid-state nanopores. (**c**) Optical micrograph of the preamplifier integrated with solid-state nanopores. (**d**) Magnified image of the preamplifier integrated with solid-state nanopores. (**e**) Optical image of a solid-state silicon nitride membrane chip mounted in the fluid cell. (**f**) Transmission electron microscope image of a silicon nitride nanopore with a diameter of 4 nm. Reproduced with permission from [119]. Copyright (2012) Springer Nature.

With the advancement of microelectronics technology and nanofabrication, solid-state nanopores (Figure 2) have gained a prominent position and can be integrated with front-end amplification chips to reduce measurement system noise [119].

## 4. Transimpedance Amplifier Design

**Figure 3:**
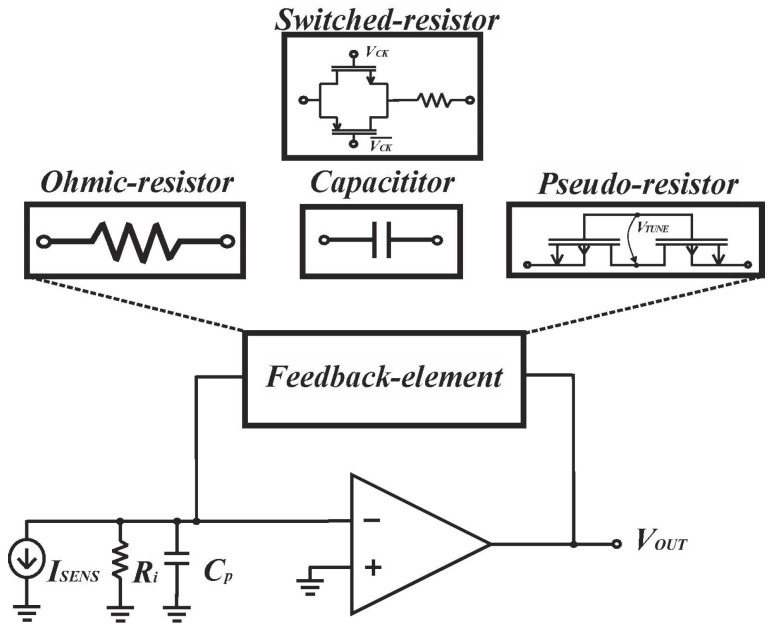
Transimpedance amplifier with different feedback elements. Reproduced with permission from [16]. Copyright (2022) Elsevier.

The versatility of feedback components in transresistance amplifier design makes the design more flexible. In this chapter, we classify transresistance amplifiers based on their feedback components (Figure 3) and present cutting-edge designs for each feedback scenario. Additionally, we provide informative insights to guide the design process.

### 4.1. Resistive Feedback

As the feedback element for TIAs, standard resistors require an immense resistance value of 1 GΩ to achieve the desired amplification factor for measurement, which not only introduces significant thermal noise but also limits the circuit bandwidth due to the inevitable parasitic capacitance and occupies a large area during chip layout.

#### 4.1.1. Pseudo-Resistor

Pseudo-resistors have established their distinct position within biomedical sensing [120,121,122] owing to their ability to provide tunable high-value resistance and serve as a highly effective alternative to ohmic resistors.

Pseudo-resistors have become an indispensable technology for circuit designers, utilizing the resistive behavior of diodes operating in weak reverse regions connected to MOS transistors. However, due to their susceptibility to process and temperature variations, engineers have proposed new methods [123,124,125,126,127,128,129] to improve pseudo-resistor designs to achieve ideal performance. For example, Djekic et al. used a pseudo-current mirror (P-CM) [130] to reduce the influence of process, voltage, and temperature (PVT) variations and connect multiple pseudo-resistor elements in series to significantly improve the linearity of the transimpedance characteristic [131]. Ultimately, the proposed improved Multi-Element Pseudo-Resistor (MEPR) was used as a feedback component for the resistive feedback TIA, achieving the limit noise level that Ohmic resistance can reach. As a result, it has become an effective method for designing high-performance TIA to meet advanced applications such as nanopore biosensing. Kim et al. [122] pointed out that the traditional pseudo-resistor method suffers from varying pseudo-resistor values when applied to nanopore current sensing due to changes in the command voltage needed to control DNA motion during sensing [132]. They proposed a gate-source voltage (V_GS_)-constant pseudo-resistor technique for constructing pseudo-resistor TIAs to address this issue. However, in the MEPR approach the resistance value is proportionally dependent on the reference resistor and is unaffected by other factors [131], which is suitable for nanopore biosensing applications.

After the emergence of multi-element pseudo-resistor technology, it has become widely used for achieving high dynamic range through tuning in preamplifier designs. In 2021, Djekic et al. [15] implemented all resistors as MEPRs in the R-TIA design with a dominant feedback capacitor. As a result, both AC and DC transimpedances were adjustable over five decades, ranging from 440 kΩ to 150 GΩ. One year later, they applied tunable multi-element pseudo-resistors (MEPRs) to an I-D-TIA design [14]. By reducing leakage current in the biasing network and implementing improved ESD protection techniques, they achieved a dynamic range of input currents spanning over five decades, as well as tuning of AC and DC transimpedances between 3 MΩ–1 GΩ and 460 kΩ–300 GΩ, respectively. Enhanced amplifiers with extended dynamic range have the potential for broader applications in biosensing.

#### 4.1.2. Switched Resistors

Switched resistors are widely used in various circuit designs for biomedical applications due to their high flexibility in resistance values and arrangements. For example, switched resistors can be used as critical components to achieve different functions in active RC filters [133], preamplifiers [134], switched resistor filters [135,136], pulse generators [137], and other circuits. In addition, due to the widespread use of CMOS technology, switched resistors are used for analog signal processing in digital circuits. For example, using switched resistors in digital-to-analog converters (DACs) can reduce the impact of reference path resistance on DAC performance [138].

However, it was not until recently that switched resistors were first applied as feedback elements in transimpedance amplifiers for biomedical applications [16]. The conventional switched resistor approach [16,135] uses a transmission gate switch (TG) and a polysilicon resistor in series, controlled by a digitally adjustable clock signal. The average current flow through the series varies with the duty cycle of the clock signal, leading to a corresponding change in the equivalent conductance of the circuit. The conductance is proportional to the duty cycle of the clock signal, enabling flexible adjustment of the circuit conductance using digital control signals. As TIA is a low-noise front-end, Centurelli et al. [16] devoted significant effort to noise optimization, particularly in minimizing the noise generated by the TG switches in the switched resistor used as a feedback element. In addition, they optimized the Operational Transconductance Amplifier (OTA) design to achieve low input noise, demonstrating lower noise and power consumption in simulations than pseudo-resistors.

In forthcoming research, the distributed switched resistor approach [135] may be deemed worthy of pursuit in TIA design. This technique can achieve high resistance multiplication factors up to several thousand even when employing minuscule duty cycles. As such, it effectively mitigates the pernicious effects of parasitic capacitances on equivalent resistance values, a paramount consideration for TIA, where enhanced gain is imperative.

#### 4.1.3. Ohmic-Resistor

Ohmic-resistor is a type of resistor that follows Ohm’s law and has a linear relationship between its resistance value and the current passing through it. This category includes poly, chip, and through-hole resistors, all of which are commonly used in circuits to provide precise, stable, and reliable resistance values. However, high resistance values can result in significant thermal noise and high parasitic capacitance. In addition, high-resistance polyresistors can occupy a large chip area, making direct integration impractical [13,131]. Nevertheless, due to their more straightforward circuit structure compared to minimizing capacitor feedback and pseudo-resistor feedback, methods such as reducing parasitic resistance and minimizing noise have been proposed to enable the application of this feedback method in biosensor interface circuits for nanopores and other applications [139,140,141].

First, Gu et al. [139] discovered that membrane capacitance amplifies noise from applied voltage, and that suppressing this noise effectively reduced noise in nanopore sensing. They developed an instrumentation system with ultra-low noise performance based on resistive feedback TIA technology which achieves a p-p noise of 3.26 pA with a 5 kHz filter during single-molecule signal recording. In addition, Yun et al. employed a novel technique called a split-resistor [140] to address the significant limitation of high parasitic capacitance resulting from using high feedback resistance on the 3 dB bandwidth. By dividing a large resistor into N parts with equal resistance, the split-resistor technique reduces the parasitic capacitance of each resistor by approximately N times. As a result, the 3 dB bandwidth increased from 70 Hz to 41 kHz while retaining the advantages of low input noise of 570 fARMS and high gain of 179.9 dBΩ offered by high feedback resistance. Moreover, Zhong et al. created a four-channel electrochemical instrument with low noise level, by combining an array of Au electrodes with amplifiers on the circuit board. Their instrument uses a two-stage amplifier with a frequency compensation circuit [142]. The first- and second-stage amplifiers provide gains of 160 dB and 20 dB, respectively; thus, the ultra-low picoampere-level current can be amplified to the millivolt level with a current gain of 180 dB, avoiding the use of GΩ-level feedback resistors, which facilitates high bandwidth and low noise performance. This cascaded amplification approach has been applied in CMOS designs as well.

The three studies discussed above were all discretely implemented. Now, we shift our focus to the use of CMOS technology. Wang et al. proposed a low-noise readout circuit with a noise amplitude as low as 11 fARMS/sq(Hz) and a bandwidth of up to 1.3 MHz [141]. The circuit can detect sub-pA range current inputs due to the novel electrostatic discharge (ESD) leakage current cancellation stage. To achieve a total transmission impedance gain of 160 dB, a preamplifier with a current gain of 40 dB is set up for amplifying the current, followed by a TIA with a 1 MΩ Poly resistor as the feedback element used as a post-stage amplifier. There has been a shift in approach to CMOS circuit design. Due to their limitations in achieving the required gain level, designers have moved away from relying solely on feedback resistors to provide necessary transresistance gain. Instead, a multi-stage amplification method has been adopted to achieve the desired total gain of the front-end amplification circuit.

Similarly, Kim et al. have proposed a low-power and high-precision interface circuit to read DNA in applications that use multiple nanopores [143]. Due to the simplicity and reliability of the hardware, they chose to use a resistive feedback TIA and a voltage gain non-inverting amplifier at the TIA’s output stage to compensate for insufficient transimpedance gain. They proposed a non-inverted structure to replace the differential amplifier in order to avoid the need for an output buffer along with a new offset cancellation module to reduce harmful input offset voltage and significantly improve accuracy. For traditional resistive feedback TIA circuits, the simplicity and stability of the hardware allow circuit designers to focus on optimizing other aspects of the circuit, making this approach more suitable for applications with special requirements.

### 4.2. Capacitive Feedback

Low noise and high signal-to-noise ratio have always been the pursuit of TIA design in biosensing, because physiological signals are easily overwhelmed by noise. Capacitive feedback elements are suitable because capacitors are noiseless components, leading to better noise performance in TIA design. Therefore, capacitive feedback is becoming increasingly popular. In the topology of capacitive feedback, the output voltage is an integration of the input current. However, capacitive feedback TIA has a significant disadvantage, namely, that the charge accumulated across the capacitor can result in output voltage saturation of the amplifier. Two techniques are commonly used to overcome this limitation. The first method is the discrete-time (DT) technique, which periodically adds a reset switch to clear the charge stored in the feedback capacitor. Another approach is the continuous-time (CT) method, which adds a DC cancellation circuit to prevent output saturation by eliminating the baseline current.

#### 4.2.1. Discrete-Time Capacitive Feedback

**Figure 4:**
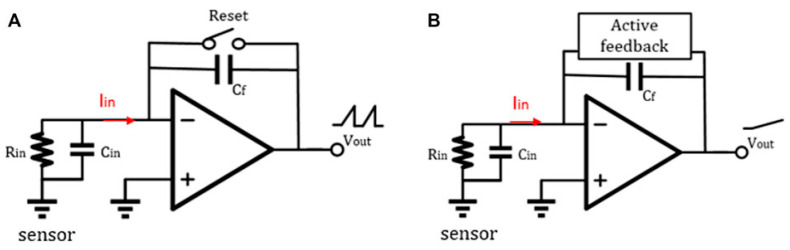
(**A**) Feedback network of discrete-time TIA and (**B**) feedback network of continuous-time TIA [144]. Reproduced with permission from Liu, Fan, Chen, Wan, Mao, and Yu, Front. Electron., published by Frontiers Media, 2022.

**Figure 5:**
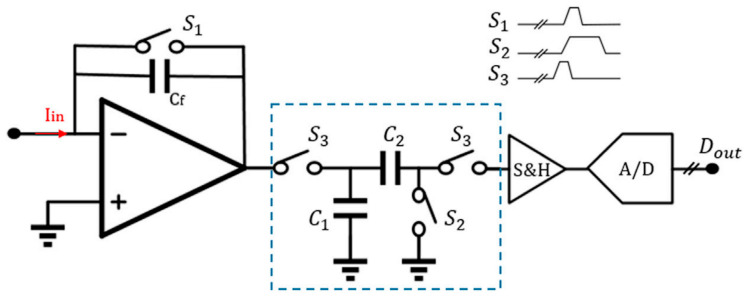
A typical front-end architecture utilizes the CDS-based discrete-time method [144]. Reproduced with permission from Liu, Fan, Chen, Wan, Mao, and Yu, Front. Electron., published by Frontiers Media, 2022.

The schematic diagram of a traditional discrete-time reset model [144] is shown in Figure 4A. Periodic resetting of the feedback capacitor is used to prevent operational amplifier saturation. However, resetting can result in data loss, particularly at lower frequencies where reset time must be minimized. Sensitivity can be improved by using a smaller feedback capacitor, typically ranging from 15–50 fF. Recently, Jiang et al. proposed an analytical model for switched-capacitor transimpedance amplifiers (SCTIAs) [145]. Their model was based on charge redistribution and designed a transimpedance amplifier (TIA) using correlated double sampling (CDS) (Figure 5) and cross-coupling techniques to optimize the noise current, power consumption, and transimpedance gain folded back to the input. The TIA achieved a transimpedance gain of 206 dB and a bandwidth of 3 kHz. The baseline noise at 1 kHz was 2.96 fA/√Hz, and the power consumption was 0.643 mW. The SCTIA model uses a fully differential amplifier and multiple logic-controlled MOS switch toggles. By controlling the timing of three pairs of switches, the saturation problem of the feedback capacitor is solved, and voltage sampling is completed. The impact of offset and flicker noise on the output can be eliminated through CDS technique in this design. The DT reset approach is typically affected by KCT noise and folded-back high-frequency noise in the front-end circuit. As a solution, the correlated double sampling (CDS) technique is implemented to enhance the readout sensitivity [146,147]. CDS works by sampling the input signal twice, with the first sample containing only low-frequency noise and offset and the second sample being the sum of the low-frequency noise and the signal.

The approach of Hsu and Hall [148] using an hourglass ADC presents a fundamentally different architecture for amplifying and digitizing signals compared to traditional DT-TIA circuits. Using an hourglass switch instead of periodically resetting the TIA feedback capacitor allows the polarity of the input signal to be reversed within a user-defined time window, contributing to higher sensitivity and linearity while eliminating the problem of saturation in the integrator stage. Moreover, the hourglass ADC achieves a high dynamic range by converting the integrator output from voltage to frequency. Therefore, this novel approach may inspire new design ideas and promote the development of more efficient and higher-performance DT-TIAs.

Achieving high throughput is critical for DNA sequencing technology. To address this need, Dawji et al. have developed an amplifier array capable of reading high-speed mixed signals using 130-nm CMOS technology [149]. This array amplifies and digitizes picoampere-range current signals. It comprises thirty identical channels that work in tandem, with each channel featuring a DT-TIA and an in-pixel SAR ADC operating with correlated double sampling (CDS). The DT-TIAs can detect nanopore currents at the picoampere level, offering a gain of G ohm, which represents the first time a complete, multiple-channel, integrated ADC array ROIC has been reported for nanopore-based DNA sequencing. Additionally, it consumes only one-tenth of the power usage of other DT-TIAs.

#### 4.2.2. Continuous-Time Capacitive Feedback

**Figure 6:**
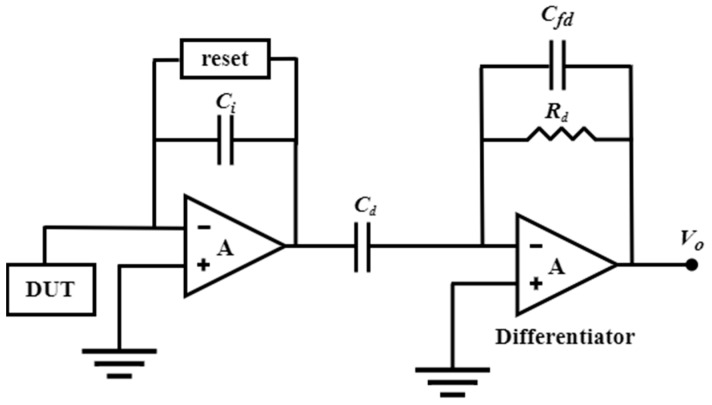
Schematic diagram of an integrator–differentiator transimpedance amplifier (I-D-TIA).

Leakage current and noise-folding of the reset switch are the primary limitations of DT-TIA, rendering it unsuitable for high-frequency applications. In contrast, the continuous-time method can be used for higher-frequency applications because it introduces an active feedback loop that solves the amplifier’s saturation problem by eliminating the baseline current at the input (Figure 4B). Ferrari et al. [13,150,151] proposed a two-stage TIA that utilizes a capacitive feedback first stage as an integrator, followed by a second stage with a differentiator to achieve an overall flat frequency response. Integrating the integrator–differentiator architecture (Figure 6) offers a sturdy conversion from current to the voltage without any penalties in terms of noise or bandwidth. Furthermore, an active feedback network plays a pivotal role as a critical building block of CT capacitive TIA, providing a low-impedance path to sink input baseline current and remove charge accumulation on the integration capacitor. Regarding the latest research advances in integrating–differentiating TIA, we previously discussed the possibility that tunable multi-element pseudo-resistors (MEPRs) could replace resistors in both DC and AC signal paths of a feedback-integrated integrating–differentiating TIA, thereby achieving better noise and speed performance. This approach overcomes the sensitivity limitation of a single MEPR and addresses the high noise issue that may exist in conventional pseudo-resistors.

**Figure 7:**
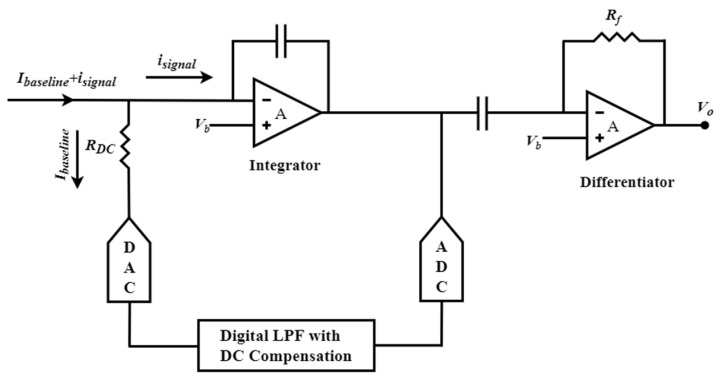
The hybrid semi-digital transimpedance amplifier (HSD-TIA) structure incorporates a noise cancellation methodology.

Hsu et al. proposed another fascinating integrator–differentiator design that employs a hybrid semi-digital (HSD) feedback network [152], as depicted in Figure 7. The feedback path comprises an ADC, digital low-pass filter, DAC, and series-connected resistor R_DC_. This innovative design accomplishes the discharge of the low-frequency component, which eliminates baseline current and attenuates flicker noise. Additionally, this network highlights a feedforward noise cancellation path that effectively eliminates voltage noise from the integrator.

**Figure 8:**
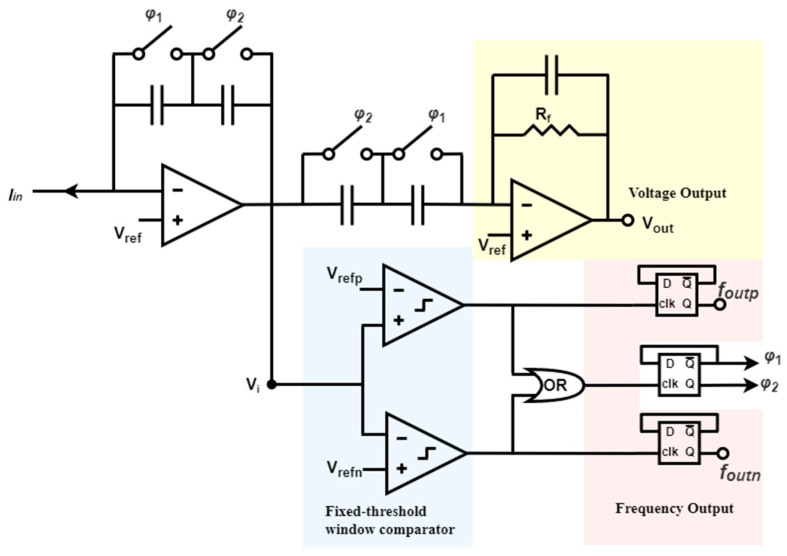
A fixed-threshold window comparator utilized in the continuous-time technique.

In another example based on continuous-time TIA, the active feedback loop uses a fixed-threshold window comparator that generates two frequency outputs and one voltage output [153], as illustrated in Figure 8. The switches φ1 and φ2 regulate the clocks and enable the self-timed switched capacitor network consisting of φ1 and φ2 to alternately match the capacitance between the integrator and differentiator stages. At any given moment, one pair of capacitors is activated while the other pair is reset, facilitating local charge balance and significantly reducing amplifier setup time. Compared to other feedback loops, this self-resetting structure does not necessitate an external clock, ensuring that system bandwidth is not restricted by predetermined sampling rates. Moreover, alternating capacitors using two switches guarantees that system resetting conserves charge and reduces the reset short and recovery time. In addition, this architecture provides an inherently and instantaneously switching method between frequency-mode and voltage-mode operations. Finally, the dynamic range is expanded by increasing the maximum measurable current. Consequently, smaller and faster current signals are made suitable for voltage output, while frequency output can represent more significant inputs.

**Figure 9:**
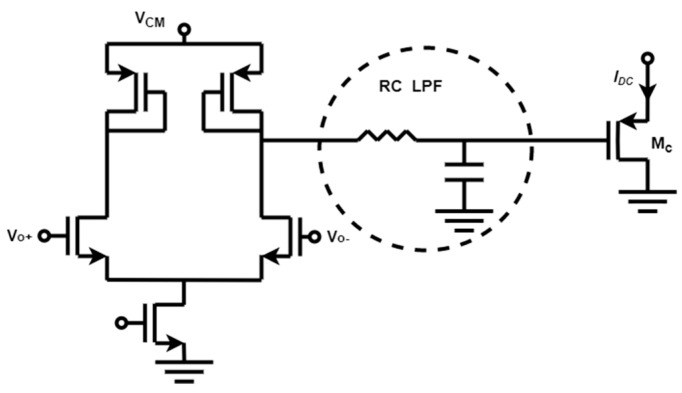
A differential circuit and low-pass filter are included in the continuous-time method.

Figure 9 shows a feedback loop that combines stability, low complexity, and robustness is established through a baseline current rejection block [17] comprised of a differential pair with diode-connected loads, followed by an RC low-pass filter, and ultimately a P-type Metal-Oxide-Semiconductor (PMOS) source follower device that receives the baseline current. This design enhances stability and minimizes both the number of poles and the loop gain in the feedback loop. Moreover, this feedback loop differentially senses the outputs (Vo+ and Vo−) of the first stage without a common-mode reference voltage, guaranteeing identical DC biases. In addition, the negative input voltage of the integrator is compensated by the diode-connected load of the first stage and the PMOS source follower Mc, thereby preventing any additional gain within the loop. Overall, this reduction in loop gain and the number of poles within the low-frequency feedback loop improves circuit stability. DC feedback loops tailored to specific needs ensure CT-TIA can reduce noise while maintaining high-frequency response.

## 5. Conclusions

This paper provides a comprehensive and informative overview of the diverse applications of nanopores in various fields, such as DNA sequencing, protein sequencing, and chiral molecule recognition. Nanopores have demonstrated their versatility and potential for breakthroughs in biosensing development. In particular, our review emphasizes the importance of understanding biological nanopores and solid-state nanopores to optimize the design of nanopore-based devices. By providing an overview of these two types of nanopores, researchers can better understand their unique properties and challenges, which can guide the selection of appropriate nanopore platforms for specific applications. Furthermore, this article has delved into the latest developments in feedback components of transimpedance amplifiers used in nanopore DNA sequencing. This section provides insights into different circuit topologies that can be employed to achieve high gain and low input reference noise. By highlighting various design schemes and circuit structures, this section equips researchers and circuit designers with the necessary tools to develop novel and efficient biomedical devices. In summary, this article is a valuable resource for those interested in the diverse applications of nanopores. The insights regarding biological and solid-state nanopores and the latest research on transimpedance amplifiers provide a foundation for further exploration and innovation in biosensing and biomedical device development.

## Data Availability

No new data were created or analyzed in this study. Data sharing does not apply to this article.
